# Books and Magazines

**Published:** 1899-11

**Authors:** 


OVER 1000 PRESCRIPTIONS, or Favorite Formulæ of Various Teachers, Authors and Practicing Physicians. The whole being carefully indexed, and including most of the newer remedies. Cloth, 300 pages, postpaid, $1.00. The Illustrated Medical Journal Co., publishers, Detroit, Mich.

This is the second edition of this handy manual, and is just from the press; it has nearly 100 pages of new matter added. As the practical worth of this kind of a book consists in its having a handy and complete index, this book has it, for some sixteen pages of small type are devoted to this object, and some of the lines have as many as twenty different references to as many different formulæ; this would go to show that there are about 2000 different prescriptions given in the volume. In other words, taking the price of the book into consideration ($1.00), it would argue that there are furnished some twenty different prescriptions for one cent. We notice that many of the newer remedies are among the prescriptions, thus bringing the treatment of many of the diseases down to date. Both old and new writers of both home and foreign countries are represented among its formulæ.

Blank pages are frequently introduced, so that a handy place is furnished for recording any new prescription that one might wish to preserve. The printed index will index all such penciled additions. if care is taken to write them opposite a page with a formulæ for similar disease; this would then save the bother of indexing the penciled additions.

PRACTICAL MATERIA MEDICA FOR NURSES. With an appendix containing Poisons and Their Antidotes, with Poison Emergencies; Mineral Waters; Weights and Measures; Dose List; and a glossary of the terms used in materia medica and therapeutics. By Emily A. M. Stoney, graduate of the Training School for Nurses, Lawrence, Mass., etc. Pages, 306. Price, $1.50, net. Philadelphia: W. B. Saunders, 925 Walnut St., 1899.

This is intended as a companion book to "Practical Points in Nursing" by the same author, and embraces in a general way the notes of a series of lectures delivered to her classes. The apothecaries' and metric systems are used, which makes the doses of drugs comprehensible to everybody. This book should become popular with nurses and those who are studying that purpose.

